# The Tri-State Medical Association of Alabama, Georgia and Tennessee

**Published:** 1889-12

**Authors:** 


### THE TRI-STATE MEDICAL ASSOCIATION OF ALABAMA, GEORGIA AND TENNESSEE.

### FIRST DAY'S SESSION.

The first annual meeting of the Tri-State Medical Association of Alabama, Georgia and Tennessee was held in Chattanooga, Tennessee, pursuant to a call to that effect, issued by the Chattanooga Medical Society. The first day's session was called to order at 10 A. M., by Dr. E. B. Wise, President of the Chattanooga Medical Society, and opened with prayer by Rev. Dr. Bochman, after which Dr. G. W. Drake delivered an eloquent address of welcome. On motion, Dr. E. B. Wise was elected temporary Chairman, and Dr. F. T. Smith was elected temporary Secretary. A Committee on By-Laws and Constitution, consisting of the following gentlemen, was appointed by the chair: Drs. Boyd of Alabama, Edge of Georgia and Barber of Tennessee.

Drs. Cowan of Tennessee, Meyers of Georgia, and Tarrent of Alabama constituted the Committee on Credentials. The meeting then adjourned to 2 P. M.

### AFTERNOON SESSION.

Meeting was called to order at 2 P. M. On motion, Dr. J. B. Cowan, of Tennessee, was unanimously elected President. The following named gentlemen were elected Vice-Presidents: First Vice-President, Dr. A. Boyd of Alabama; Second Vice-President, Dr. Edge of Georgia; Third Vice-President, Dr. Barber of Tennessee.

Dr. B. Stewart, of Chattanooga, was elected Treasurer; and Dr. Frank Trester Smith, to whom is largely due the credit of organizing the Association, was unanimously elected Secretary.

The first paper before the Association was that presented by Dr. Wm. L. Gahagan of Chattanooga: "The Physiology of the Heart and its Valves." Dr. Gahagan's paper was an able one, and after giving a *résumé* of the physiology of the heart, he proceeded to give the results of a series of experiments extending over a period of several years. This paper gave rise to an interesting discussion, by Drs. G. W. Drake, James E. Reeves and J. B. Cowan. Doctor James E. Reeves read a paper on "The Importance of the Microscope in the Practice of Medicine." Numerous cases were cited, showing how necessary is a knowledge of microscopy to the general practitioner. Drs. W. C. Townes, G. A. Baxter and J. B. Cowan discussed this paper at some length.

Dr. J. E. Purdon, of Alabama, reported a case of "Fracture of the Skull in an Old Man—Recovery." The fracture was an extensive one and was situated over the third frontal convolution. Aphasia was persistent, and though the patient has recovered, he still finds it difficult to pronounce names of persons and places. This paper elicited considerable discussion from Drs. J. L. Lynch, G. A. Baxter, E. T. Camp, James E. Reeves, W. L. Gahagan and W. B. Wells. At this juncture telegrams were read from Dr. Battey of Rome, Ga., and Drs. Howell and McRae of Atlanta, Ga., regretting their inability to be present, and expressing their sympathy and hearty co-operation in the movement to organize a Tri-State Medical Association. Adjourned until 8 P. M.

EVENING SESSION.

Dr. Andrew Boyd, of Scottsboro, Ala., presented a paper on "Croup and Pneumonia," which was thoroughly discussed by Drs. P. D. Sims, W. L. Gahagan, E. T. Camp, J. E. Purdon, Cooper, J. F. Lynch and Reeves. This was followed by an interesting article on "Imaginary Foreign Bodies in the Throat," by Dr. Max. Thorner of Cincinnati, Ohio, discussed by Drs. Steele, Cooper and Smith. Adjourned to meet at 9:30 A. M.

SECOND DAY'S SESSION.

Dr. J. B. Cowan called the meeting to order, opening it with prayer. An Auditing Committee, composed of Drs. G. A. Baxter, E. T. Camp and R. J. Trippe, was appointed. Drs. Rothwell, Gahagan and Townes composed the Committee on

Arrangements for the next annual meeting. The following sections were created and Chairman elected:

State Medicine—P. D. Limes, M. D., Tennessee.

Psychological Research—J. E. Purdon, M. D., Alabama.

Otology—R. D. Boyd, M. D., Alabama.

Practical Microscopy—Jas. E. Reeves, M. D., Tennessee.

Opthalmology—N. C. Steele, M. D., Tennessee.

Obstetrics—W. T. Blackford, M. D., Georgia.

Gynecology—R. J. Trippe, M. D., Tennessee.

Materia Medica and New Remedies—J. F. Lynch, M. D., Chattanooga.

Surgery—G. A. Baxter, M. D., Tennessee.

Experimental Physiology—W. L. Gahagan, M. D., Chattanooga.

Laryngology—Max Thorner, M. D., Cincinnati, Ohio.

Practice—G. W. Drake, M. D., Tennessee.

Dr. J. A. Long, of Athens, Tenn., read an able paper on "Typhoid Fever," based upon an experience of over twenty-five years and the treatment of five hundred cases. The position taken by Dr. Long, in dealing with the treatment of this disease, was very conservative, and the results which he has obtained prove the value of little medication and proper nursing. Dr. G. W. Drake strongly advocated the use of quinine both as a food and an antipyretic. This feature in the treatment of typhoid fever was the source of a spirited discussion. Dr. J. T. Lynch strongly condemned the use of quinine in typhoid fever, for antipyrine and antifebrin, when carefully watched, are safer and far more efficient antipyretics than quinine; he further stated, that as to giving quinine as a food he could imagine nothing more thoroughly disagreeable to the palate of a sick person. Dr. Purdon, of Alabama, stated that he had seen a case in a child of one year old. Dr. Gahagan could not see how such a thing could occur, as Peyer's patches, the seat of the initial lesion, were not developed then. This paper was further discussed by Dr. James E. Reeves, who insisted upon the bacteriological origin of typhoid fever, and by Drs. Cooper, R. D. Boyd, E. T. Camp, C. Holtzclaw and J.

B. Cowan. Dr. Frank Trester Smith exhibited a specimen of intestinal perforation due to typhoid fever. Adjourned until 2 P. M.

### AFTERNOON SESSION.

After the meeting was called to order, Dr. W. C. Townes presented a paper on "Hypnotism and Suggestibility," which was followed by a paper on "The Sphygmograph as a New Re-agent in Psychical Research," by Dr. J. E. Purdon. These papers elicited considerable discussion, especially as to the dangers of hypnotism from a moral standpoint, and in regard to the existence of a thought wave. Dr. C. Holtzclaw then reported "Two cases of Laparotomy," followed by a paper on "Laparotomy with a Report of an Interesting Case," by Dr. J. F. Lynch, of Chattanooga. Dr. Lynch cleverly stated the indications for laparotomy, and his remarks on this subject were very profitable.

These papers were discussed by Dr. R. J. Trippe, who reported a laparotomy for an incised wound of the intestine, and by Drs. Townes, Baxter, Cowan and Green.

### EVENING SESSION.

The last paper to be presented to the Association was a "Report of a case of Persistent Pupillary Membrane," by Dr. Frank Trester Smith, which was discussed at some length by Dr. Max Thorner, N. C. Steele and G. A. Baxter.

A membership of over a hundred was announced and the meeting adjourned to meet in Chattanooga, Tenn., October 21, 1890.

